# Supplementary material for: Actionable Molecular Alterations Are Revealed in Majority of Advanced Non-Small Cell Lung Cancer Patients by Genomic Tumor Profiling at Progression after First Line Treatment
Source: Cancers (Basel). 2021 Dec 28;14(1):132. doi: 10.3390/cancers14010132 (PMC8749927; doi:10.3390/cancers14010132)
Supplement: Supplementary file 1 [file cancers-14-00132-s001.zip › Supplementary Table S2.pdf]

Modalities	Procedures, *n* (%)	Complications, *n* (%)
**Cytological biopsy modality**	**97** (74)	0 (0)
Ultrasound-/fluoroscopy-guided TTNA-FNA (lung)	17 (18)	**2** (12)
EBUS-TBNA	32 (33)	**3** (9)
EUS/EUS-B-FNA	20 (21)	0 (0)
Percutaneous FNA (non-lung)	19 (20)	0 (0)
Thoracocentesis	5 (5)	**1** (20)
Bronchoscopy: bronchial wash	4 (4)	0 (0)
**Histological biopsy modality**	**34** (26)	0 (0)
Ultrasound-guided cutting needle biopsy	11 (32)	0 (0)
Bronchoscopic forceps biopsy	17 (50)	**1** (6)
CT-guided cutting needle biopsy	5 (15)	**1** (20)
Lymph node dissection	1 (3)	0 (0)
**Total**	**131 (100)**	**8** (6)
**No. of modalities**	**Patients, *n* (%)**	**Complications, *n* (%)**
1	60 (65)	6 (10)
2	25 (27)	1 (4)
3	7 (8)	0 (0)
**Total**	**92 (100)**	**7 (8)**

Supplementary Table S2: *The numbers of procedures of each biopsy modality including the number of complications are illustrated (upper part). Each patient could have more than one biopsy-modality performed as illustrated in the lower part.*

Abbreviations: TTNA = transthoracic needle aspiration. FNA= fine-needle aspiration biopsy. EBUS-TBNA = endobronchial ultrasound transbronchial needle aspiration biopsy. EUS/EUS-B = endoscopic ultrasound of para-gastroesofagal structures/performed with EBUS-endoscope.
