# Supplementary figures and images for: Actionable Molecular Alterations Are Revealed in Majority of Advanced Non-Small Cell Lung Cancer Patients by Genomic Tumor Profiling at Progression after First Line Treatment
Source: Cancers (Basel). 2021 Dec 28;14(1):132. doi: 10.3390/cancers14010132 (PMC8749927; doi:10.3390/cancers14010132)

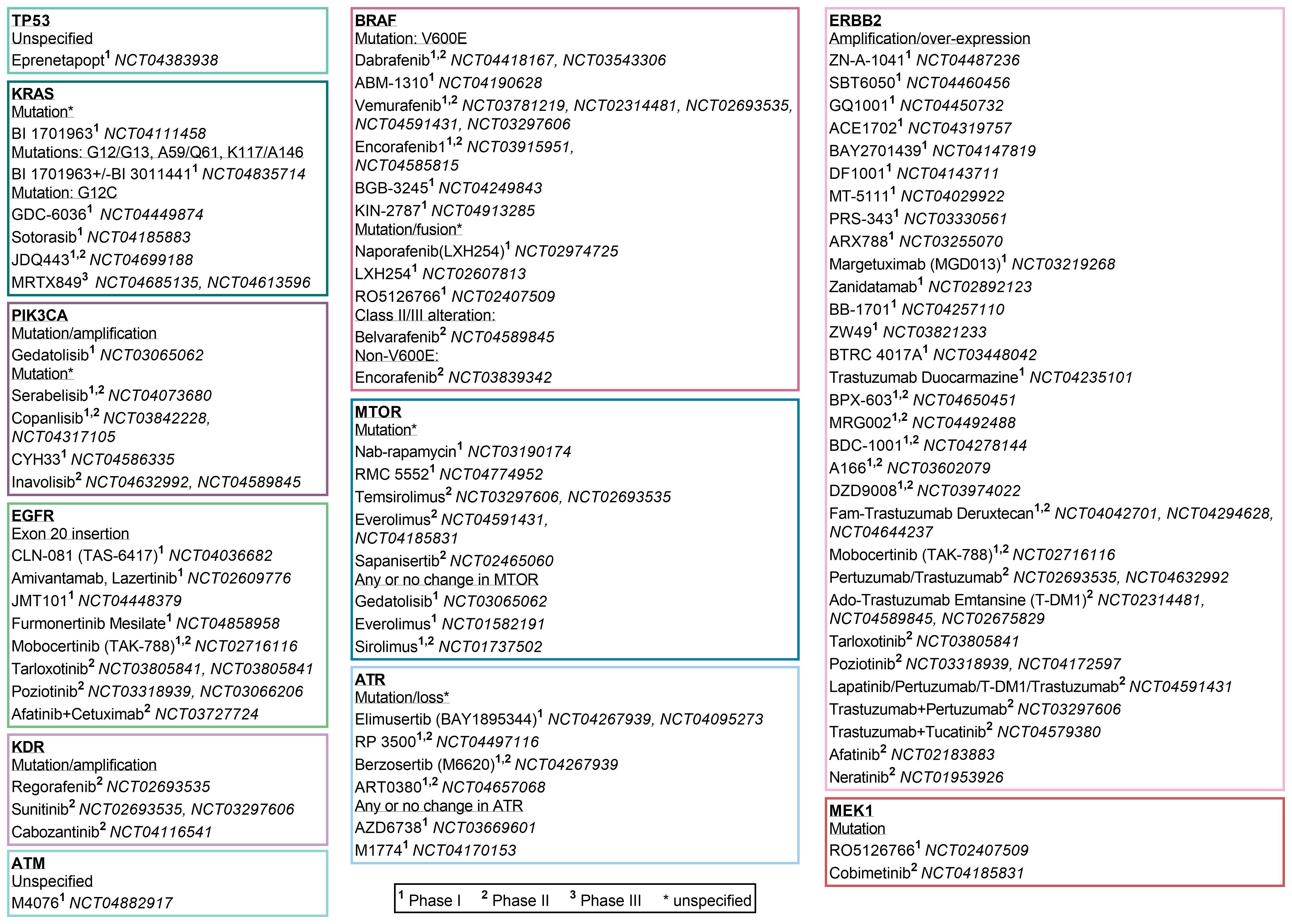

Supplement: Supplementary file 1 [file cancers-14-00132-s001.zip › Supplementary Figure S1.jpg]

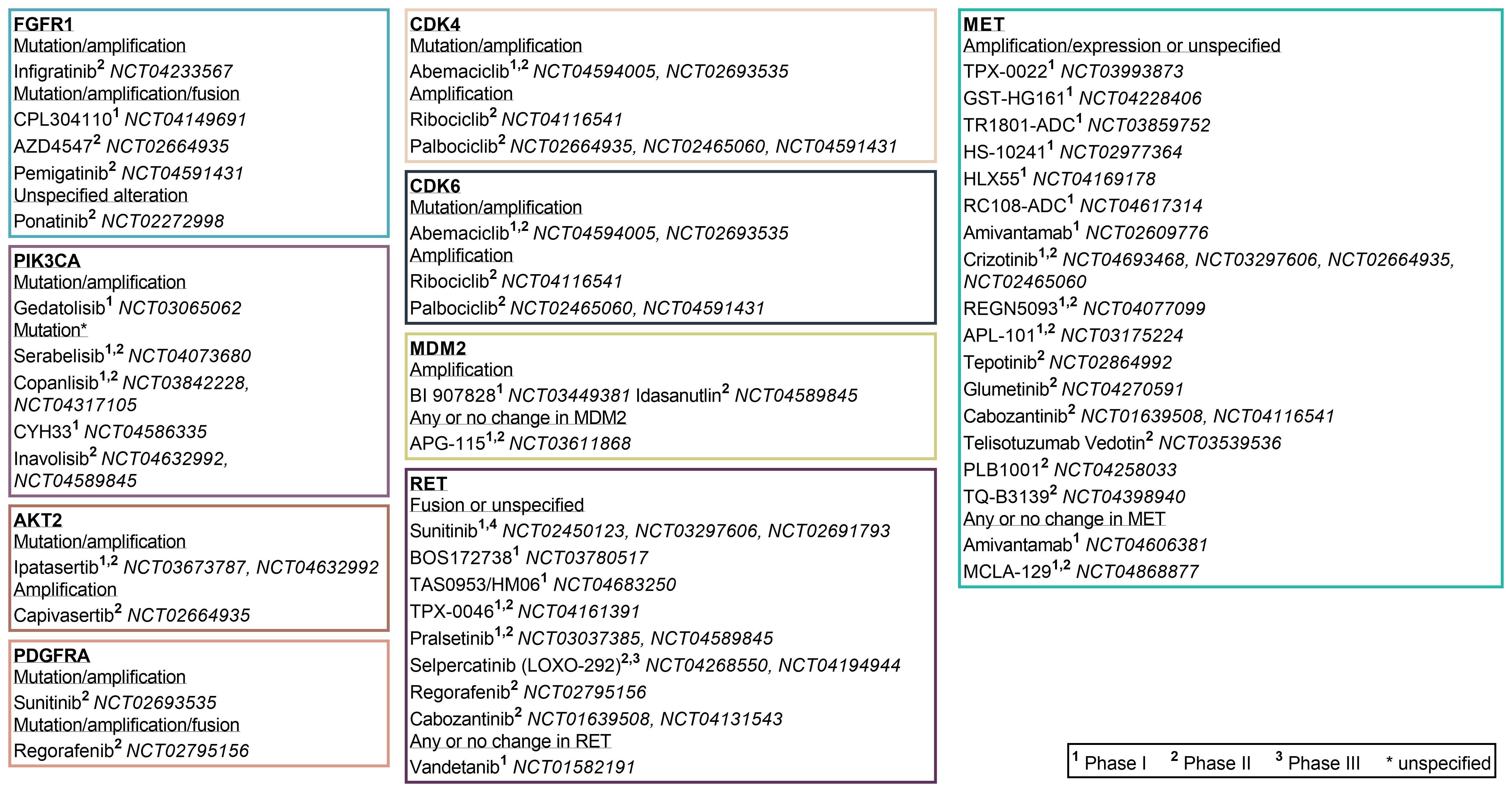

Supplement: Supplementary file 1 [file cancers-14-00132-s001.zip › Supplementary Figure S2.jpg]
